# Scalene and sternocleidomastoid activation during normoxic and hypoxic incremental inspiratory loading

**DOI:** 10.14814/phy2.14522

**Published:** 2020-07-29

**Authors:** Nada Basoudan, Antenor Rodrigues, Alessio Gallina, Jayne Garland, Jordan A. Guenette, Babak Shadgan, Jeremy Road, W. Darlene Reid

**Affiliations:** ^1^ Department of Physical Therapy University of British Columbia (UBC) Vancouver BC Canada; ^2^ College of Health and Rehabilitation Sciences Princess Nourah bint Abdulrhaman University Riyadh Saudi Arabia; ^3^ Physical Therapy University of Toronto Toronto ON Canada; ^4^ Centre of Precision Rehabilitation for Spinal Pain (CPR Spine) School of Sport, Exercise and Rehabilitation Sciences College of Life and Environmental Sciences University of Birmingham Birmingham UK; ^5^ Faculty of Health Sciences Western University London ON Canada; ^6^ Centre for Heart Lung Innovation UBC and St. Paul's Hospital Vancouver BC Canada; ^7^ International Collaboration on Repair Discoveries Vancouver BC Canada; ^8^ Division of Respiratory Medicine Department of Medicine University of British Columbia (UBC) Vancouver BC Canada; ^9^ KITE Toronto Rehab Toronto ON Canada; ^10^ Interdepartmental Division of Critical Care Medicine University of Toronto Toronto ON Canada

**Keywords:** electromyography, muscle fatigue, respiratory muscles

## Abstract

The purpose of this study was to examine scalene (SA) and sternocleidomastoid (SM) activation during normoxic (norm‐ITL; FIO_2_ = 21%) and hypoxic (hyp‐ITL; FIO_2_ = 15%) incremental inspiratory threshold loading (ITL). Thirteen healthy participants (33 ± 4 years, 9 female) performed two ITL tests breathing randomly assigned gas mixtures through an inspiratory loading device where the load was increased every two minutes until task failure. SA and SM root mean square (RMS) electromyography (EMG) were calculated and expressed as a percentage of maximum (RMS_%max_) to reflect muscle activation intensity. Myoelectric manifestations of fatigue were characterized as decreased SA or SM EMG median frequency during maximum inspiratory pressure maneuvers before and after ITL. Dyspnea was recorded at baseline and task failure. Ventilatory parameters and mouth pressure (Pm) were recorded throughout the ITL. SA,RMS_%max_ and SM,RMS_%max_ increased in association with ITL load (*p* ≤ .01 for both). SA,RMS_%max_ was similar between norm‐ITL and hyp‐ITL (*p* = .17), whereas SM,RMS_%max_ was greater during the latter (*p* = .001). Neither SA nor SM had a decrease in EMG median frequency after ITL (*p* = .75 and 0.69 respectively). Pm increased in association with ITL load (*p* < .001) and tended to be higher during hyp‐ITL compared to norm‐ITL (*p* = .05). Dyspnea was similar during both conditions (*p* > .05). There was a trend for higher tidal volumes during hyp‐ITL compared to norm‐ITL (*p* = .10). Minute ventilation was similar between both conditions (*p* = .23). RMS,_%max_ of the SA and SM increased linearly with increasing ITL. The presence of hypoxia only increased SM activation. Neither SA nor SM presented myoelectric manifestations of fatigue during both conditions.

## Graphical abstract

Sternocleidomastoid activation was greater during hypoxic than mildly normoxic incremental inspiratory loading. Scalene activation was similar during both conditions. Both sternocleidomastoid and scalene demonstrated significant increases in activation that were linearly related to respiratory loading during both hypoxic and normoxic ITL. Neither scalene nor sternocleidomastoid exhibited changes in EMG median frequency or absolute voluntary respiratory strength.

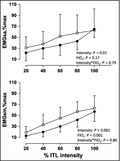

## 1 INTRODUCTION

To mimic a condition of increased ventilatory effort in the presence of hypoxemia, numerous investigators have examined the effect of hypoxia on respiratory muscle fatigue during whole‐body exercise. Studies have shown greater diaphragm fatigue development during hypoxic compared with normoxic exercise (Babcock et al., 1985; Verges, Bachasson, & Wuyam, 2010; Vogiatzis et al., 2007). Despite the considerable advancement in hypoxic exercise research in humans, there are limited data on the contribution of the inspiratory neck muscles (e.g., scalenes [SA] and sternocleidomastoid [SM]) during increased respiratory demands due to hypoxia. The only published study that compared the magnitude of neck inspiratory muscle activity was performed during hypoxic hyperpnea and showed a significant effect of hypoxia on increasing SM activation (Katayama et al., 2015).

Hypoxemia and increased work of breathing are common findings in patients with respiratory diseases (Bateman et al., 2018; Boulet et al., 2019; Spruit et al., 2013). In these populations, increased reliance on neck inspiratory muscles, such as the SA and SM, have been described to overcome increased respiratory demands (De Troyer & Boriek, 2011). However, whether this finding is due to the increased load owing to lung and/or airway pathophysiological adaptations, the presence of hypoxemia, or muscle morphophysiological adaptations is not completely understood due to the overlapping of these pathophysiological manifestations in many patients. Therefore, applying these two stimuli in a cross‐over design, would allow us to gain insights on the mechanisms used by the respiratory system to overcome each of these two different types of pathophysiological consequences of respiratory diseases. Applying this model on healthy participants, allows us to quantify the impact on hypoxia and increased work of breathing in the absence of systemic and respiratory alterations that are seen in patients with respiratory diseases (e.g., muscle morphology alterations, airflow obstruction involving both the lung or chest wall) (Bateman et al., 2018; Boulet et al., 2019; Orozco‐Levi, 2003). Therefore, the purpose of this study was to compare the magnitude of activation and myoelectrical manifestations of fatigue in the SA and SM muscle during normoxic and hypoxic incremental inspiratory threshold loading (norm‐ITL and hyp‐ITL, respectively) in healthy adults. We hypothesized that the increase in inspiratory loads would result in increased SA and SM activation and secondly, that myoelectrical manifestations of fatigue and dyspnea would be greater in the hyp‐ITL condition.

## 2 METHODS

### 2.1 Subjects

Thirteen healthy adults (9 female) aged 20 to 65 years were included. Elite athletes, active smokers, people with hypersensitive skin, and individuals who were diagnosed with respiratory diseases or chronic illnesses were excluded. All participant provided written informed consent prior to participating. The study was approved by the University of British Columbia Ethics Review Board (H14‐00952) and the Vancouver Coastal Health Research Institute (V14‐00952).

### 2.2 Experimental procedure

This study was a single‐blind cross‐over, repeated measures design. Participant attended a preliminary visit and two testing visits. During the preliminary visit, participant's anthropometric data were obtained, followed by routine spirometry as per established guidelines (Miller et al., 2005) and reference values were utilized for interpretation (Hankinson & Odencrantz, 1999). Participant performed a maximum inspiratory maneuvers (MIP) after full expiration to residual volume against occluded inspiration (by closing the valve on the inspiratory threshold loading device) according to standard technique (Black & Hyatt, 1969; Laveneziana et al., 2019) and were expressed as a percentage of predicted values (Evans & Whitelaw, 2009). Patients were familiarized with the incremental ITL protocol using a threshold trainer device (Threshold®IMT, Vacumed, Respironics Inc). For the next two testing visits, participants were asked to refrain from strenuous exercise for at least 48 hr and from caffeine intake for 12 hr prior to the ITL test. Participants performed an incremental ITL test while breathing a randomly assigned gas mixture with 21% (norm‐ITL) or 15% oxygen (hyp‐ITL) balanced with nitrogen. Gas flowed through a tube from the gas cylinder to a nondiffusing gas reservoir bag (30 L Hans Rudolph, Kansas City, MO, USA) and then via a wide bore tubing (27 mm) to the chamber of the threshold loading device. Participant were blinded to the gas composition. Visits were separated by at least 4 days. Dyspnea and peripheral oxygen saturation (SpO_2_) were quantified before and at the ITL peak using the modified 0–10 category‐ratio Borg scale (Borg, 1982) and a pulse oximetry device (Passport 2, Datascope Corp), respectively. MIP was recorded before, immediately after and 10 min after ITL for quantifying changes in MIP and its related EMG median frequency pre‐to‐post ITL as indicators of muscle fatigue. MIP maneuvers were performed as defined by international guidelines (Laveneziana et al., 2019) and the highest value of three reproducible maneuvers is reported. Ventilatory parameters were measured continuously throughout both tests.

### 2.3 Inspiratory pressure threshold loading protocol

Participants were comfortably seated in an upright position with the head supported in a neutral position on a head‐chin rest. A custom‐built ITL device, as described previously, was used (Basoudan, Shadgan, Guenette, Road, & Reid, 2016; Shadgan, Guenette, Sheel, & Reid, 2011). Wearing nose clips, participants breathed the gas mixture through the mouthpiece for four minutes before the ITL began. The ITL started by adding a 100‐g weight to the ITL device. Thereafter, the load was increased by 50 g every two minutes until task failure as previously described (Basoudan et al., 2016; Shadgan et al., 2011). Task failure was defined as the point when the subject could not generate enough inspiratory pressure to raise the plunger on two consecutive breaths (Basoudan et al., 2016; Shadgan et al., 2011). Participants were instructed to pace their breathing frequency by targeting auditory cues of 2 s inspiration and 4 s expiration for a 33% duty cycle and a breathing frequency (fb) of 10 breaths/min (Basoudan et al., 2016; Shadgan et al., 2011). No other instructions were given regarding breathing pattern such that lung volumes were variable among participants (Basoudan et al., 2016; Shadgan et al., 2011).

### 2.4 Ventilatory parameters

Participants breathed through a flanged mouthpiece that was connected to a two‐way nonrebreathing valve (1400, Hans Rudolph). The valve was connected to a pneumotach (3813, Hans Rudolph) between the threshold loading device and the inspiratory port of the two‐way nonrebreathing valve (Hans Rudolph) to measure inspiratory flow, which was subsequently used to determine *f*b, tidal volume (V_T_), and minute ventilation (V_E_). Continuous measures of inspiratory mouth pressure (Pm) and partial pressure of end‐tidal CO_2_ (P_ET_CO_2_) were recorded from a port close to the mouthpiece via a pressure transducer (MP45, Validyne Corp) and carbon dioxide analyzer (Model 17630, VacuMed), respectively. Ventilatory parameters were sampled at 200 Hz and converted to digital signals (PowerLab 16/30, ADInstruments, Colorado Springs, CO). The pressure‐time‐product (PTP) was determined as the product of the integration of Pm during the inspiratory flow and *f*b. Before each experiment, standard volume, pressure, and gas calibration procedures were performed. Ventilatory parameters were averaged as the last 30 s of each ITL quintile of duration.

### 2.5 Respiratory muscle activity (EMG)

EMG signals were acquired with high‐density surface EMG with two electrode arrays (ELSCH 008, OT Bioelettronica) from the sternal head of the SM and anterior SA. The two electrode arrays, which consisted of eight electrodes (1 mm diameter with 5 mm interelectrode distance), were attached to the skin with adhesive foam **(**KITAD008, OT Bioelettronica) and were filled with conductive cream (CC1, OT Bioelettronica). Skin preparation was performed according to SENIAM recommendations (Hermens, Freriks, Disselhorst‐Klug, & Rau, 2000). The identification of the SM was determined by palpating the muscle belly during submaximal neck flexion contractions, and the electrode array was placed on the lower half of the muscle. For the SA, the electrode array was placed in the posterior triangle of the neck at the level of the cricoid cartilage. Both arrays were placed along the approximate muscle fiber orientation. Two reference electrodes (H59P, Kendall‐LTP, Covidien) were placed on the lateral aspect of the right acromion and coracoid processes. The EMG signals were collected in monopolar modality using OT BioLab (OT Bioelettronica), amplified with a gain of 500 and sampled at 2,048 Hz.

### 2.6 EMG data processing

EMG data were processed using MATLAB (MATLAB, version 8.3.0. Natick, Massachusetts: The MathWorks Inc. 2014). To increase the detection volume along the approximate muscle fiber direction, single differentials were calculated from nonadjacent monopolar signals. Signals were filtered (Butterworth, 4th order, 20–400 Hz) and visually inspected; noisy channels were excluded from the analysis. For each array, the three consecutive differential channels with the largest EMG amplitude were included in the analysis. This approach helped avoid the channels above the innervation zones. Root mean square amplitude and median frequency were calculated on nonoverlapping epochs of 250 ms and averaged across the three channels. The ITL test duration was divided into five equal quintiles (20%, 40%, 60%, 80%, and task failure). For each quintile, SA and SM root mean square was measured throughout norm‐ and hyp‐ITL and normalized by its maximum activation during MIP maneuvers (RMS_%max_) as a measure of the intensity of muscle activation. MIP were measured according to standard technique (details above). The average of the last five inspirations of each quintile is reported. SA and SM EMG median frequency were analyzed during MIP maneuvers pre, immediately and 10 min after ITL and compared between norm‐ and hyp‐ITL. Significant decreases in EMG median frequency before versus after ITL would be considered as myoelectric manifestations of muscle fatigue (Falla, Jull, Hodges, & Vicenzino, 2006; Falla, Rainoldi, Merletti, & Jull, 2003; Marco, Alberto, & Taian, 2017).

### 2.7 Statistical analysis

Data are expressed as mean ± *SE*. Statistical analysis was performed with SPSS (version 22.0, SPSS Inc, Chicago, IL). Normality in data distribution was assessed using the Shapiro‐Wilk test for each cell of the design, and homogeneity of variances was assessed by Levene's test. Variables that were not normally distributed were log‐transformed before carrying out further analysis but were reported in its natural units to facilitate interpretation. A two‐way ANOVA was conducted to examine the main and interaction effects of the inspired oxygen fraction (FiO_2_) and ITL intensity on the SA and SM RMS_%max_, EMG median frequency and ventilatory variables (Statistics, 2017). Statistical significance was defined as *p* < .05.

## 3 RESULTS

### 3.1 Descriptive characteristics

All participants had spirometry, body mass index, and MIP within the expected normal range (Table 1). The maximum ITL loads (norm: 481 ± 43 and hyp: 481 ± 42 grams) and test times (norm: 17 ± 2 and hyp: 17 ± 2 min) were similar at task failure during both norm‐ and hyp‐ITL. Dyspnea increased significantly from zero at baseline to 8 ± 2 and 7 ± 2 during norm‐ITL and hyp‐ITL, respectively, (*p* < .001 for both) but the increase was similar between conditions (*p* > .05). SpO_2_ was significantly lower at peak hyp‐ITL compared to norm‐ITL (94 ± 1% vs. 98 ± 1, respectively; *p* = .005).

**TABLE 1:** Sample characteristics

	Mean ± SE
Age, yrs	33 ± 4
BMI, kg^.^m^−2^	23.0 ± 1
FVC, %pred	90 ± 4
FVC, L	4.7 ± 1.5
FEV_1_, %pred	97 ± 5
FEV_1_/FVC, %pred	101 ± 3
MIP, cmH_2_O	123 ± 10
MIP, %pred	100 ± 4

Abbreviations: BMI, body mass index; FEV_1_, forced expiratory volume in the first second; FVC, forced vital capacity; MIP, maximum inspiratory pressure.

### 3.2 Respiratory muscle EMG during normoxic or hypoxic ITL

**FIGURE 1:**
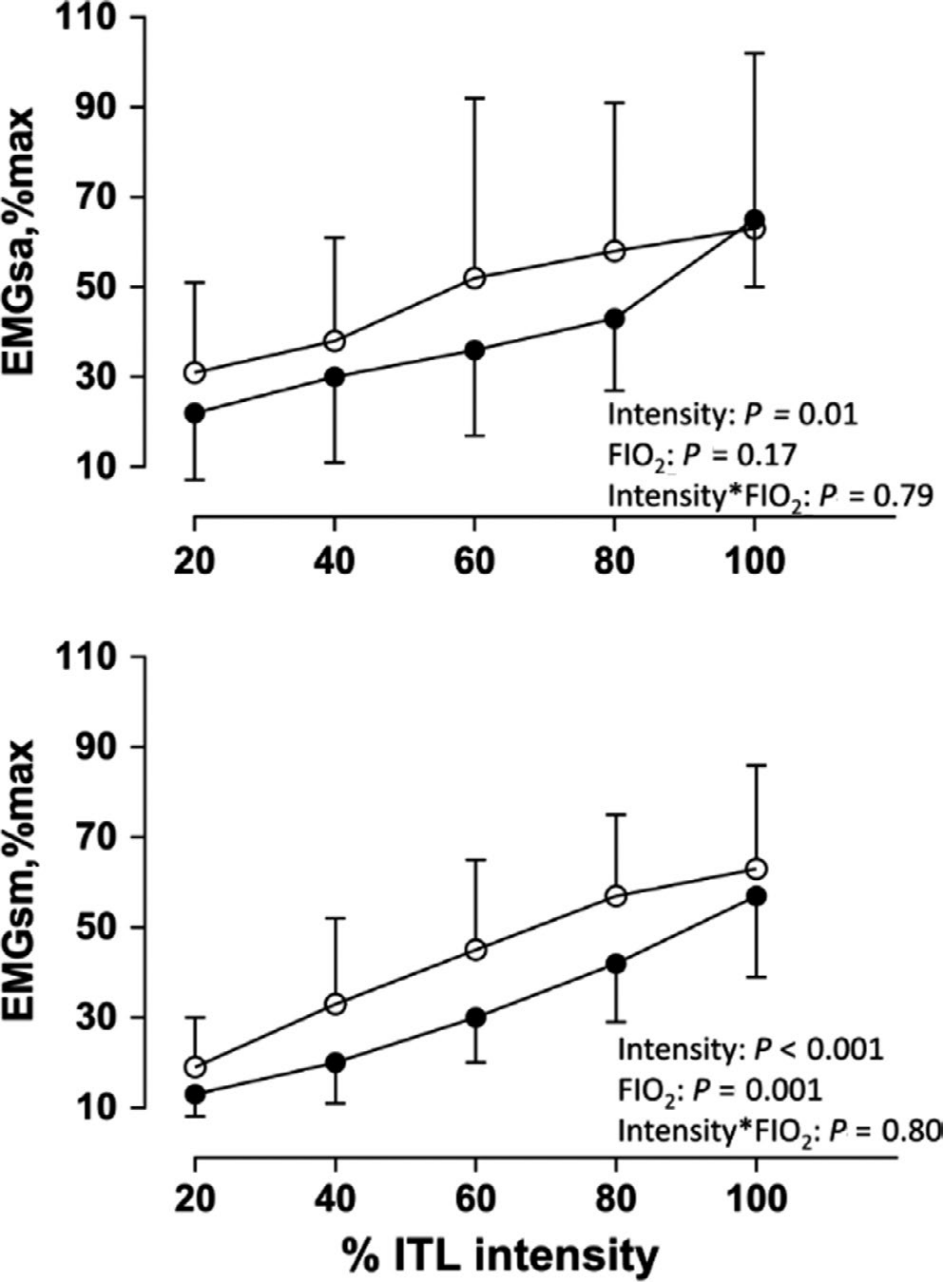
Scalene (SA) and sternomastoid (SM) root mean square in % maximum activation (RMS,%max) during normoxic (closed circles) and hypoxic ITL (open circles). Intensity: two‐way ANOVA main effect for ITL intensity; FIO_2_: two‐way ANOVA main effect for norm‐ITL vs. hyp‐ITL; Intensity*FIO_2_: two‐way ANOVA interaction between ITL intensity and FIO_2_

There was no statistically significant interaction effect between ITL intensity and FiO_2_ on SA,RMS_%max_ (*p* = .79) nor SM,RMS_%max_ (*p* = .80). However, SA,RMS_%max_ and SM,RMS_%max_ increased in association with increasing ITL intensities (*p* = .01 and *p* < .001, respectively; Figure 1). SA,RMS_%max_ was similar between norm‐ and hyp‐ITL (*p* = .17) but SM,RMS_%max_ was greater during hyp‐ITL compared to norm‐ITL (*p* = .001; Figure 1).

There was no statistically significant interaction effect between ITL intensity and FIO_2_ on SA (*p* = .91) or SM EMG median frequency (*p* = .78) during MIP assessed pre‐and post‐ITL and during recovery. Changes in SA and SM EMG median frequency were similar between norm‐ and hyp‐ITL (*p* = .52 and 0.41, respectively; Figure 3). SA (*p* = .75) and SM EMG median frequency (*p* = .69) were similar during MIP pre, post, and 10 min after ITL.

### 3.3 Breathing pattern, ventilation, and MIP values during normoxic and hypoxic ITL

There were no interaction effects between ITL intensity and FiO_2_ for Vt (*p* = .99), V_E_ (*p = *.96), Pm (*p = *.84), PTP (*p = *.70), and P_ET_CO_2_ (*p* > .99). Vt and V_E_ did not significantly increase in association with increasing ITL intensities (*p* = .83 and 0.74 respectively). However, P_ET_CO_2_, Pm, and PTP did increase in association with increasing ITL intensities (Figure 3; *p* < .001 for all). Although the increase in V_E_ was similar during hyp‐ and norm‐ITL (*p = *.23), there were trends for greater Vt (*p* = .10), Pm (*p = *.05) and PTP (*p = *.11) during the former. P_ET_CO_2_ was greater during hyp‐ITL compare to nor‐ITL (*p = *.02). MIP values were statistically similar before, immediately and 10 min after norm‐ITL (116 ± 10, 119 ± 11, 113 ± 10 cmH_2_O, respectively; *p* > .05) and hyp‐ITL (111 ± 11, 115 ± 11, 113 ± 11 cmH_2_O, respectively; *p* > .05). Likewise, changes in MIP before and post ITL were similar in both the normoxic and hypoxic conditions (*p* > .05).

## 4 DISCUSSION

The unique findings of the study are that SM activation (RMS_%max_) was greater during hypoxic than mildly normoxic incremental ITL; however, SA activation was similar during both conditions. Of note, both SM and SA demonstrated significant increases in activation that were linearly related to respiratory loading during both hyp‐ITL and norm‐ITL. Despite the increased levels of activation in SA and SM, neither muscle exhibited changes in EMG median frequencies during MIP maneuvers or changes in absolute MIP values that would indicate muscle fatigue. Measures of peak load, dyspnea scores, and ventilatory variables did not differ at task failure between norm‐ and hyp‐ITL; however, Vt tended to be higher during hyp‐ITL. Therefore, these results only partially support our initial hypothesis that hyp‐ITL would evoke greater SA,RMS_%max_ and SM,RMS_%max_, as well as greater dyspnea scores, and that norm‐ and hyp‐ITL would generate myoelectrical manifestation of fatigue in both SA and SM.

SM EMG has been evaluated during different breathing patterns in healthy individuals and patients with respiratory disease. In healthy young adults, the SM does not appear to be recruited during resting breathing or low‐intensity ITL (Chiti et al., 2008; Nobre et al., 2007). However, SM,RMS_%max_ has consistently shown to be increased when higher loads are imposed on the respiratory system (De Troyer & Boriek, 2011; Katayama et al., 2015; Rodrigues et al., 2019; Shadgan et al., 2011). An increase in SM,RMS_%max_ during hypoxic hyperpnea compared to normoxic hyperpnea has been described (Katayama et al., 2015). Although hyperpnea and ITL provide different mechanical and physiological stimuli to the respiratory muscles (Rodrigues et al., 2019), our findings show that the same pattern of increase in SM,RMS_%max_ was present during ITL. Also in patients with chronic obstructive pulmonary disease (COPD), the SM demonstrated greater activation during constant‐load ITL at 30% (Andrade et al., 2005) or 50% (Rodrigues et al., 2019) of MIP, as well as during resting hyperpnea (Rodrigues et al., 2019). Increased SM,RMS_%max_ has also been described in patients that failed a spontaneous breathing trial (Parthasarathy, Jubran, Laghi, & Tobin, 1985). Therefore, our results are consistent with previous findings in different populations that show increased SM activation as a reserve to overcome increasing demands imposed to the respiratory muscles (De Troyer & Boriek, 2011; Katayama et al., 2015; Rodrigues et al., 2019; Shadgan et al., 2011) ‐ not only tidal volume, but also Pm and PTP tended to be higher under the hyp‐ITL.

**FIGURE 2:**
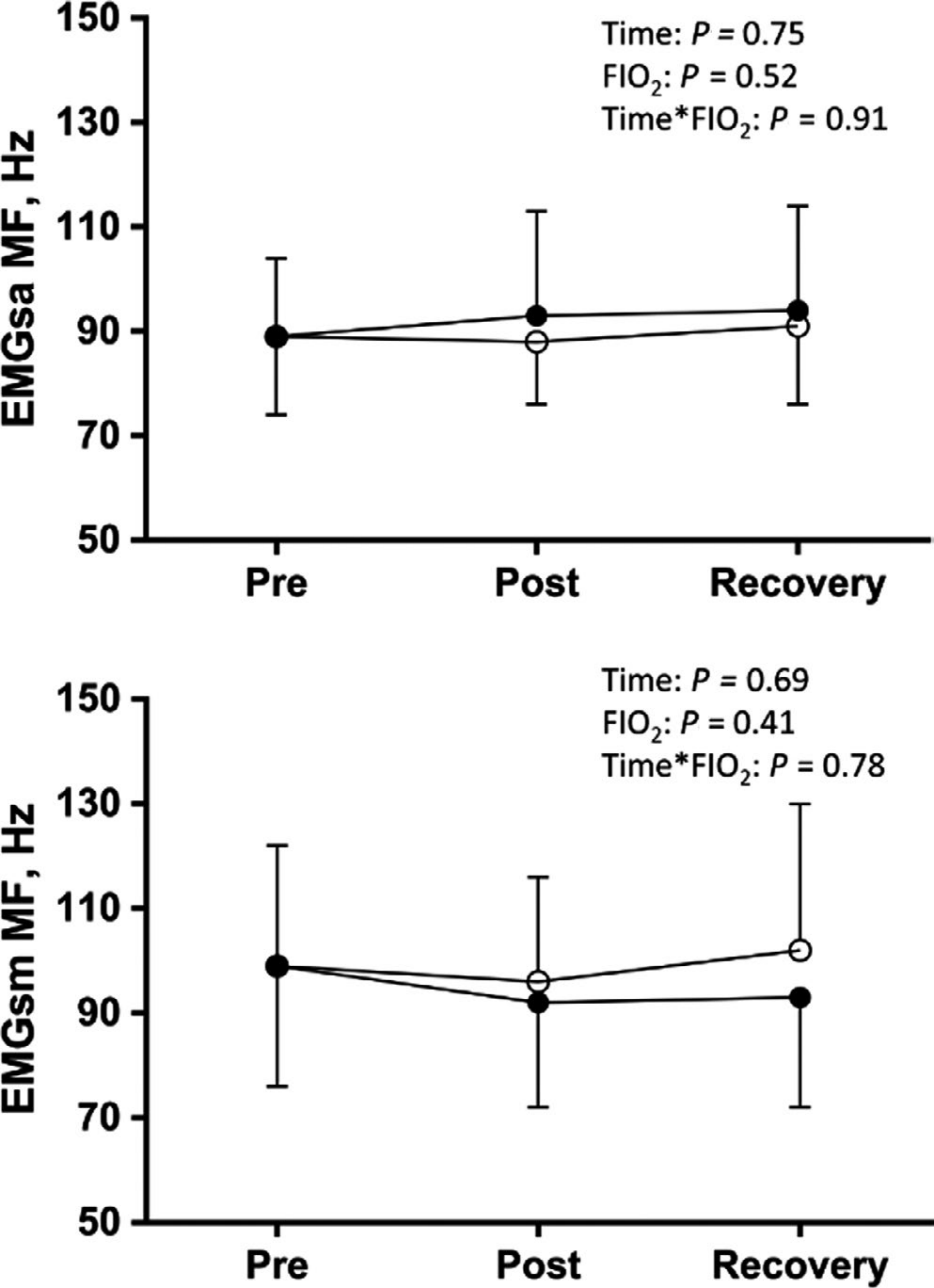
Scalene (SA) and sternomastoid (SM) electromyography (EMG) median frequency (MF) during maximum inspiratory pressure (MIP) maneuvers pre, post, and recovery (10 min after ITL) during normoxic (closed circles) and hypoxic ITL (open circles). Intensity: two‐way ANOVA main effect for ITL intensity; FIO_2_: two‐way ANOVA main effect for norm‐ITL vs. hyp‐ITL; Intensity*FIO_2_: two‐way ANOVA interaction between ITL intensity and FIO_2_

**FIGURE 3:**
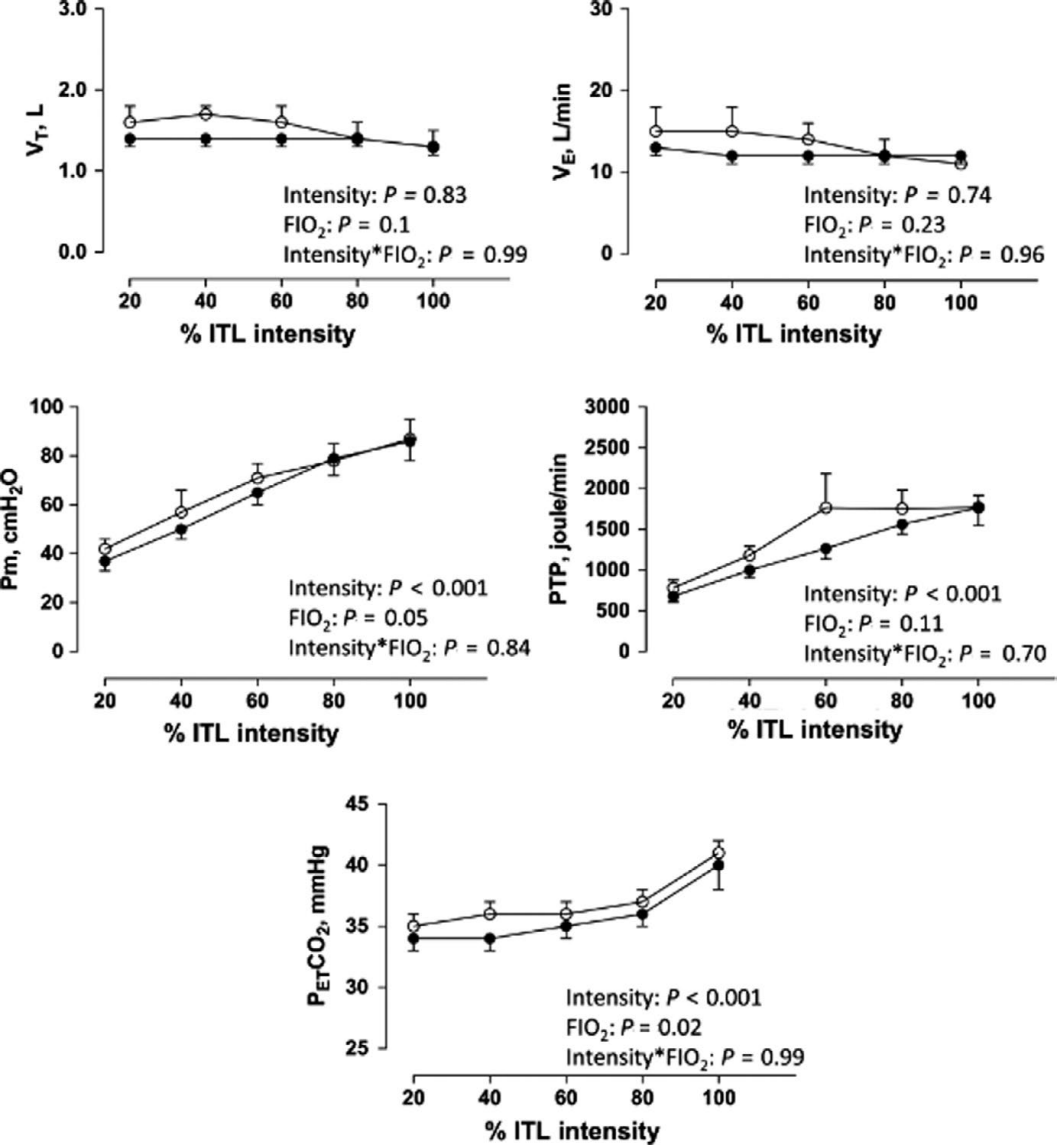
Tidal volume (V_T_), minute ventilation (V_E_), mouth pressure (Pm), pressure‐time‐product (PTP), and end‐tidal CO_2_ (P_ET_CO_2_) during normoxic (closed circles) and hypoxic ITL (open circles). Intensity: two‐way ANOVA main effect for ITL intensity; FIO_2_: two‐way ANOVA main effect for norm‐ITL vs. hyp‐ITL; Intensity*FIO_2_: two‐way ANOVA interaction between ITL intensity and FIO_2_

The greater activation of the SM during increased ventilatory demands has been attributable to its length‐tension relationship at higher lung volumes or its ability to generate stronger and faster contractions compared to other inspiratory muscles, including the diaphragm (De Troyer & Boriek, 2011; Farkas, 1991; Farkas & Rochester, 1985). The SA, however, is a primary inspiratory muscle that is active even during tidal breathing (De Troyer & Boriek, 2011). The absence of a statistically significant increase in SA,RMS_%max_ during the hyp‐ITL may reflect a preferable strategy of reliance on increasing activation of accessory inspiratory muscles, one of which is the SM. Hence, the increased load imposed by the ITL (Figure 3) is shared amongst both accessory and primary inspiratory muscles and protects both against fatigue development (Figure 2). This fact is further supported by greater variability in SA,RMS_%max_ during hyp‐ITL (Figure 1). It may suggest that although some participants did increase SA activation during hyp‐ITL, this strategy was not consistent in our entire sample.

The lack of decrease in SA and SM median frequency suggest that no muscle fatigue was present (Figure 2). This conjecture is strengthened by similar MIPs before and after ITL. Previous studies successfully described myoelectric manifestations of fatigue of SM and SA in people with and without neck pain (Falla et al., 2003), and reduced myoelectric manifestations of fatigue after endurance training (Falla et al., 2006). In these studies, however, SM and SA were tested as neck flexors during sustained isometric tasks. The absence of myoelectric manifestations of fatigue in respiratory muscle observed in this study supports the postulate that inspiratory muscle fatigue is dependent on breathing frequency and duty cycle. Previous observations have shown that although breathing against a constant resistive load at a duty cycle of 70% may induce diaphragm fatigue, shorter duty cycles as we used herein (i.e., 33%) do not appear to induce similar effects (Sheel et al., 2001; St Croix, Morgan, Wetter, & Dempsey, 2000). The long exhalation period and controlled breathing frequency might have prevented fatigue development even during mild hypoxic ITL (Figure 2). Another factor that may have contributed to a lack of fatigue in the current experiment is that, besides breathing frequency, breathing pattern was not controlled in our study. Thus, the recruitment pattern of other inspiratory muscles may have had prevented the development of SA and SM muscle fatigue as well (Laghi et al., 2014; Ramsook et al., 2016). Nevertheless, the fatigue of the SM and SA has been described previously during moderate‐intensity (i.e., 50%MIP) constant load ITL until task failure (Derbakova et al., 2020). Differences in total test load ensuing to test design (incremental vs. constant load) and duration (mean of 38 min vs. 17 min in our study) may, at least in part, explain these differences. Collectively, our findings suggest that task failure occurred because of factors besides SA or SM muscle fatigue.

Task failure has been described as a defensive mechanism, protecting muscles against contractile fatigue and damage during exercise. Hence, several theories have attempted to explain the underlying contributing factors to task failure. One of which is that task failure may occur due to hypoventilation coincident with hypercapnia. Hypoventilation can occur as an inhibitory reflex to limit further respiratory muscle recruitment and possible injury (Laghi et al., 2014). In our study, a significant increase in P_ET_CO_2_ was noted in association with increasing ITL intensities (Figure 3). Furthermore, P_ET_CO_2_ was also higher during hyp‐ITL compared to norm‐ITL while ventilation was similar (Figure 3). These findings are in agreement with those who reported that hypercapnia can contribute to task failure (Gorman, McKenzie, & Gandevia, 1999; Laghi et al., 2014; McKenzie, Allen, Butler, & Gandevia, 1985; Roussos & Koutsoukou, 2003). Hypercapnia has also been linked to increased dyspnea sensation (Banzett et al., 1990; Banzett, Lansing, Reid, Adams, & Brown, 1989; Gigliotti, 2010; Manning & Schwartzstein, 1995). In fact, hypercapnia was associated with intolerable dyspnea that could contribute to task failure (Gorman et al., 1999; Rohrbach, Perret, Kayser, Boutellier, & Spengler, 2003). Likewise, dyspnea has also been linked to the magnitude of the SA,RMS_%max_ and SM,RMS_%max_ (Chiti et al., 2008; Schmidt et al., 2013). Of note, dyspnea scores were high (7–8 out of 10) and similar at peak norm‐ and hyp‐ITL. Taken together, one can suggest that increased P_ET_CO_2_ and greater SM,RMS_%max_ likely contributed to greater dyspnea perception and, together, led to participants’ task failure during both conditions.

### 4.1 Clinical implications and further hypotheses

The strategy of increasing the SM,RMS_%max_ to a greater extent during hyp‐ITL while SA,RMS_%max_ was increased to a lesser extent corroborate the role of the SM as a reservoir that is called upon when the respiratory system confronts increased demands (De Troyer & Boriek, 2011; Rodrigues et al., 2019). Furthermore, it also supports the notion of “sharing” the load amongst different respiratory muscles according to their biomechanical and morphological specificities (De Troyer & Boriek, 2011). In this way, multiple respiratory muscles may be used as a functional unit in an attempt to avoid respiratory muscle fatigue (Rodrigues et al., 2019; Tobin, Laghi, & Jubran, 2012). This hypothesis needs, however, further investigation. Although in our study participants could “choose” to stop before muscle fatigue was developed, possibly due to increasing dyspnea sensation. This option is not be possible for people with chronic or acute lung diseases when increased loads are relentlessly imposed. In this case, persistent hypercapnia and greater inspiratory muscle activation could likely induce respiratory muscle fatigue and respiratory failure.

### 4.2 Methodological strengths and limitations

The association between the respiratory muscle load of the ITL and the hypoxemic condition resembles, to a certain extent, what is noted in many different lung conditions such as COPD, interstitial lung diseases, and critically ill patients. While the ITL resembles and even exaggerates the increased work of breathing that is derived by altered pulmonary mechanics in lung disease, inhalation of a hypoxic gas mixture induces hypoxemia that might parallel that induced by ventilation‐perfusion mismatching and limited diffusion capacity. High‐density surface EMG using linear arrays has been validated to detect and to improve the quality of myoelectrical activity recordings from the neck muscles in healthy and diseased populations (Falla et al., 2003, 2006; Marco et al., 2017). This technique can limit the influence of anatomical factors, such as the presence of an innervation zone under the electrodes, on the EMG estimates (Smith et al., 2015). Also, fatigue‐related decreases in median frequency have been demonstrated to be more prominent when assessed over many electrodes than in simulated bipolar detection (Gallina, Merletti, & Vieira, 2011). Thus, these attributes strengthen the validity of our finding of no shift in EMG median frequency.

Limitations of this study included the absence of additional respiratory muscle EMG measurements, including the crural diaphragm that is considered the gold standard for measures of respiratory drive. Measurement of multiple respiratory muscles during our experiments would enable further investigation of how the respiratory muscles are activated as a functional unit instead of examining individual contributions. The fact that we measured myoelectric manifestations of fatigue must also be acknowledged in light of the absence of the gold standard of phrenic nerve stimulation with changes in twitch Pdi (transdiaphragmatic pressure). Possible cross‐contamination between SA and SM electrical activity must be acknowledged as well. However, the small EMG electrode size and interelectrode distance and the different patterns of activation observed suggest that this probably had a minimal impact on our data. Assessing respiratory effort with a Borg scale in addition to dyspnea scores would have provided additional information regarding the main reason for participants to stop the test. Due to the test profile (i.e., increasing respiratory load), one would expect respiratory effort to contribute to task failure as well. Although we have shown in a previous study that ITL associated with a hypoxic gas mixture with 15% oxygen evoked greater respiratory muscle deoxygenation, we acknowledge that the absence of this measurement limits our ability to investigate whether greater respiratory muscle activation was due to greater muscle hypoxia, greater respiratory muscle load, or a combination of both. Likewise, lower hypoxic gas mixtures (e.g, 10% oxygen) have been used previously and would have provided a greater physiological stress. The absence of SpO_2_ measurements through the ITL hinders our ability to investigate its continuous changes during the test. The sample used in this study presented a wide age, from 20 to 65 years old. Future studies should investigate whether age contributes or modifies outcomes.

In conclusion, SA and SM activation intensity increased linearly with increasing ITL intensities, but only the SM activation increased when breathing a mild hypoxic gas mixture.

## CONFLICT OF INTEREST

The authors declare that they have no conflict of interest to disclose.

## AUTHOR CONTRIBUTIONS

NB and WDR contributed to the conception and design of the study. NB, AR, AG, JG, JAG, BS, JR, and WDR contributed to data acquisition, analysis, and/or interpretation of the data, and draft of the manuscript. All authors revised and approved the submitted version of the manuscript.
